# Vervet monkey (*Chlorocebus pygerythrus*) behavior in a multi-destination route: Evidence for planning ahead when heuristics fail

**DOI:** 10.1371/journal.pone.0198076

**Published:** 2018-05-29

**Authors:** Julie Annette Teichroeb, Eve Ann Smeltzer

**Affiliations:** 1 Department of Anthropology, University of Toronto Scarborough, Toronto, Ontario, Canada; 2 Department of Anthropology, University of Toronto, Toronto, Ontario, Canada; University of Sussex, UNITED KINGDOM

## Abstract

Animal paths are analogous to intractable mathematical problems like the Traveling Salesman Problem (TSP) and the shortest path problem (SPP). Both the TSP and SPP require an individual to find the shortest path through multiple targets but the TSP demands a return to the start, while the SPP does not. Vervet monkeys are very efficient in solving TSPs but this species is a multiple central place forager that does not always return to the same sleeping site and thus theoretically should be selected to find solutions to SPPs rather than TSPs. We examined path choice by wild vervets in an SPP experimental array where the shortest paths usually differed from those consistent with common heuristic strategies, the nearest-neighbor rule (NNR–go to the closest resource that has not been visited), and the convex hull (put a mental loop around sites, adding inner targets in order of distance from the edge)–an efficient strategy for TSPs but not SPPs. In addition, humans solving SPPs use an initial segment strategy (ISS–choose the straightest path at the beginning, only turning when necessary) and we looked at vervet paths consistent with this strategy. In 615 trials by single foragers, paths usually conformed to the NNR and rarely the slightly more efficient convex hull, supporting that vervets may be selected to solve SPPs. Further, like humans solving SPPs, vervets showed a tendency to use the ISS. Paths consistent with heuristics dropped off sharply, and use of the shortest path increased, when heuristics led to longer paths showing trade-offs in efficiency versus cognitive load. Two individuals out of 17, found the shortest path most often, showing inter-individual variation in path planning. Given support for the NNR and the ISS, we propose a new rule-of-thumb termed the “region heuristic” that vervets may apply in multi-destination routes.

## Introduction

Foraging animals move between multiple food sources throughout the day and should be selected to maximize their food intake rate [1,2]. If resources are equally rewarding, it is expected that natural selection should have favored the ability to find the shortest path among food sites, as this leads to maximum energy gains with the least energy output [3]. However, finding the shortest path through a set of targets is not a simple task. This is analogous to famously difficult combinatorial optimization problems, like the Traveling Salesman (or Salesperson) Problem (TSP) or the Shortest Path Problem (SPP) (i.e., optimal Hamiltonian path problem or open-TSP), where the number of possible routes increases exponentially as targets are added [3–5]. In a TSP, an individual must find the shortest path through a set of targets before returning to the start, completing a full tour [4]. While in a SPP, the individual need not return to the original location, leaving open the choice of which site to visit last and leading to different optimal routes [3]. The type of optimization problem an animal species would have been selected to solve may depend upon their foraging strategy. Central place foragers, that return to the same nest, burrow, or sleeping site each night [6,7], should theoretically be selected to solve the TSP, since they do a tour of their range each day. Alternatively, multiple central place foragers, which are species that have several different sleeping locations within their home range [8], would benefit most from strategies that help solve the SPP.

Research has shown that animals often choose paths between locations that are close to optimal in terms of distance [9,10–15] but it has been difficult to determine their decision-making processes given the difficulty of solving the TSP and the SPP [11,16,17]. Every day, people also move through multiple destinations and rather than spending time and energy calculating the best paths, humans generate fast and relatively accurate solutions using simple heuristics [5]. Cognitive heuristics are “rules-of-thumb” that evolved because they can quickly and easily provide reasonable solutions to everyday problems [18]. Two of the most common heuristics humans report using for multi-destination route problems are the nearest-neighbor rule (NNR—choosing the closest site that has not been visited) and the convex hull heuristic (placing a mental loop around all sites to be visited and tightening, sequentially including inner points in order of distance) [5,19,20]. The NNR has been used to explain the behavior of foraging bighorn sheep [21], the convex hull has been used to explain the way vervets solve a multi-destination route [11], and a heuristic based on feeding patch size agrees with hummingbird foraging [22]. Even small-brained insects are quite efficient in their foraging routes and recently it has been shown that a heuristic model agreed with the establishment of traplines by bumblebees, though bees take multiple trials, improving slowly over time, before arriving at efficient routes [16,17]. Thus, like humans, other animals also likely approximate solutions to routing problems using relatively simple heuristics.

Teichroeb [11] has previously shown that wild vervet monkeys (*Chlorocebus pygerythrus*) at Nabugabo choose paths that are consistent with several heuristics, including the NNR and convex hull, but this study was unable to differentiate between taking the shortest path and the convex hull in most instances. This is an issue because vervets have been shown to have the greatest capacity to solve TSP-like problems of the three nonhuman primates tested (including chimpanzees and yellow-nosed monkeys [12,13]). Cramer and Gallistel [9] showed that captive vervets chose the shortest possible path among six food sites and appeared to consider the location of at least two further goals before choosing their route, seemingly planning three-steps ahead. However, this study had a small sample size of individuals (*n* = 4) and trials (*n* = 26) and Janson [3] has suggested that the apparent success of vervets in this study may be explained by them avoiding the researchers, who were standing near the array, during testing. Thus, the question remains as to whether vervets do solve multi-destination routes frequently or whether they are applying a heuristic strategy.

The Teichroeb [11] study also brought up an additional question. Vervet monkey groups typically have several sleeping sites (EAS, JAT, unpubl. data) and thus they are multiple central place foragers [8]. This leads to the assumption that if vervets are selected to solve multi-destination routes, they should be selected to solve SPPs rather than TSPs. In the SPP presented to the vervets in Teichroeb [11], they most often used paths consistent with the convex hull, even though this is not an ideal heuristic to solve SPPs. The convex hull often optimally solves TSPs because these are closed loops and while it can be used for SPPs when individuals begin and end at nearby points [23], it is unlikely to have been selected for in animals, like vervet monkeys, that are multiple central place foragers. Much less research has been directed towards how humans solve SPPs, compared to TSPs. Even though they are not ideal in SPPs, we may apply similar strategies as we do to TSPs. For instance, MacGregor and colleagues [24] found that in open versions of the TSP, people still used the convex hull most often, as well as the NNR, and they avoided crossing their paths. However, these studies require subjects to choose their paths on paper where all targets are visible and two-dimensional, which is quite different and likely easier than choosing an efficient path when moving through the real world, which is nonuniform, dynamic, and three-dimensional (for example, [25]), and where the vestibular system and path integration become involved [26]. It is known that when people are trying to find the shortest path to a distant destination they tend to minimize their mental effort by making the fewest turns possible [27,28]. This tendency causes people to choose straight paths disproportionately at the start of their routes, leading to different paths when moving from destination A to destination B, than when moving from B to A [27,29]. Bailenson and colleagues have termed this the Initial Segment Strategy (ISS) [30].

**Fig 1:**
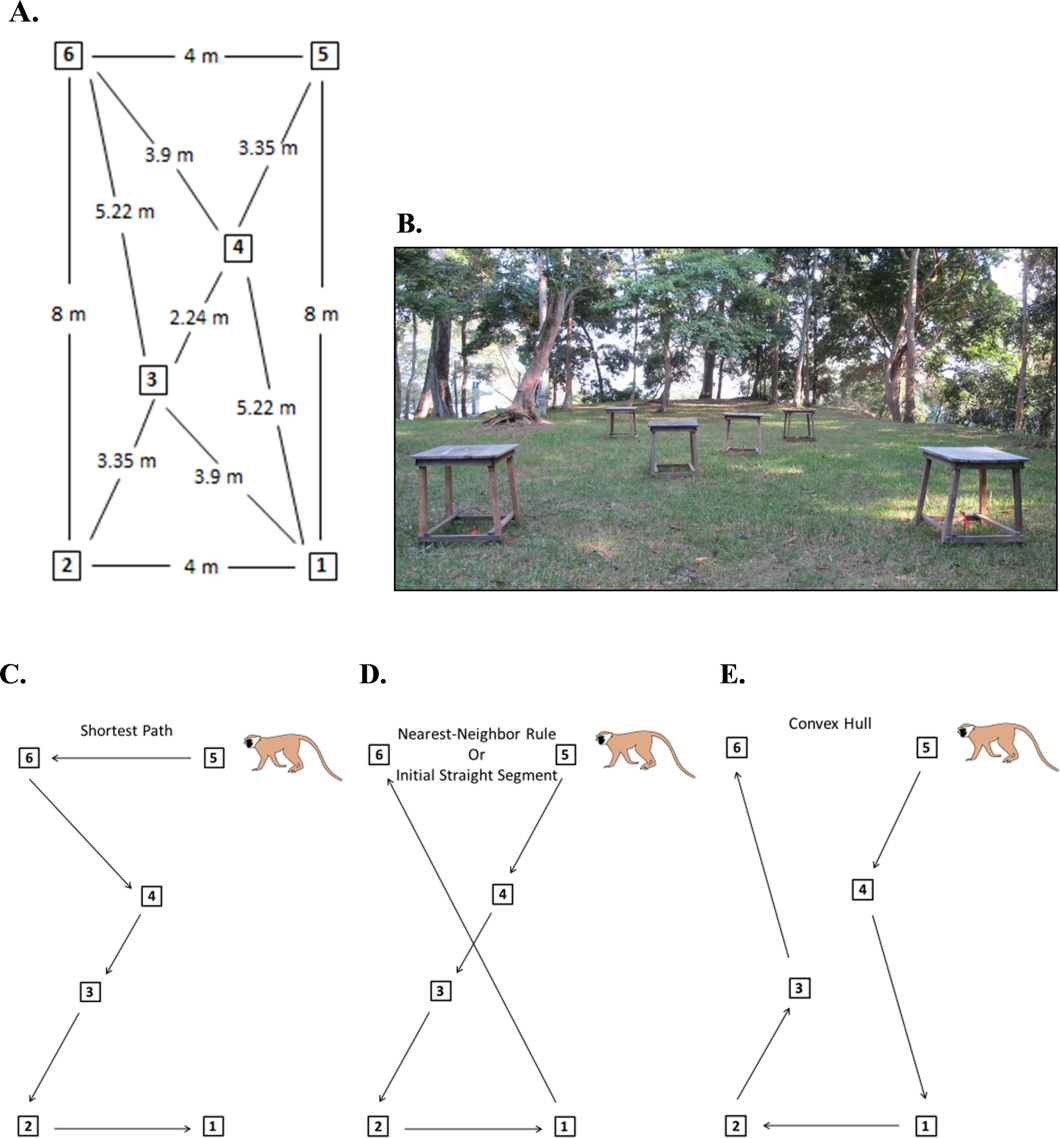
Z-shaped experimental platform array provided to wild vervet monkeys at Lake Nabugabo, Uganda. (**A**) shows the exact distances from the center of each platform and (**B**) shows how the array looked in the field. (**C**) shows the shortest path when starting at platform 5, while (**D**) shows the path that conforms to the nearest-neighbor rule. Paths consistent with the initial segment strategy could occur from platform 5 and platform 2 and left open the order of the last two platforms. (**E**) shows a path consistent with the convex hull from platform 5.

In this study, we aimed to explore further how vervet monkeys (*Chlorocebus pygerythrus*) solve multi-destination routes and solve the two questions that remained from the Teichroeb [11] study. Namely, 1) can vervets find the shortest path through a multi-destination route, and 2) will they still use paths consistent with the convex hull in a SPP where the geometry of platforms is not conducive to it? Further, 3) we sought to determine whether, like humans, vervets avoided turning until necessary, using paths consistent with an ISS. We designed a Z-shaped experimental array with six destinations (Fig 1A) to test the same group of wild vervets at Lake Nabugabo, Uganda previously used by Teichroeb [11]. In this Z-array, the shortest path was always different from paths consistent with the NNR, the NNR was always different from the convex hull, and the shortest path was different from the convex hull from all but the two central starting points. Thus, this array allowed us to determine how often the shortest path was actually found by the vervets versus when paths were consistent with some heuristic. In addition, the Z-array provided a clear SPP to the monkeys because it was less circular then the array presented in Teichroeb [11], thus it was less likely to favor use of the convex hull as a solution, helping to determine if the monkeys do apply this solution (that actually favors solving TSPs) regardless of route geometry. Finally, the Z-array contained a straight segment that allowed us to look at whether vervets favored straight paths at the start of a route (i.e., the ISS, [30]), something that may be more beneficial in solving SPPs rather than TSPs.

We placed feeding platforms in the Z-array within a clearing in the study group’s home range (Fig 1B) and each was baited with the same small reward. Some individuals would travel ahead of the group to complete trials alone, while in other cases food competition occurred because multiple individuals were present. Here, we only analyze trials where single, identified foragers went through the array alone. We hypothesized that if vervet monkeys solve SPPs without the use of heuristics, they should choose the shortest path most often. Alternatively, if they have been selected to use heuristics that are better suited to solving SPPs than TSPs while foraging, they should choose paths consistent with the NNR and ISS more often than the shortest path and more often than the convex hull.

## Materials and methods

We carried out a multi-destination route choice experiment with one group of wild vervet monkeys (*Chlorocebus pygerythrus*) at Lake Nabugabo, in the Masaka Region of Uganda (0°22’-12°S and 31°54’E) from August 10 to September 6, 2015 (28 days). Vervets are small-bodied cercopithecine monkeys that are widespread throughout their range and considered least concern by the IUCN [31]. The study group (M group) contained 28 individuals (2 adult males, 9 adult females, 2 subadult males, 3 subadult females, 12 juveniles and infants) that were individually recognizable by features of the face and body (Table 1). One peripheral adult male also followed the group and participated in experiments (VP).

**Table 1:** Age-sex class and sample size of trials for the 17 individual vervet monkeys in the data set.

Individual ID	Age-Sex Class	Trial Sample Size
JK	Adult male	40
PY	Adult male	171
VP*	Adult male	98
GR	Adult female	31
LP	Adult female	72
LT	Adult female	3
MA	Adult female	44
PT	Adult female	12
RM	Adult female	4
TB	Adult female	29
TS	Adult female	17
DG	Subadult female	34
PG	Subadult female	9
TG	Subadult female	38
GA	Juvenile male	7
PF	Juvenile male	1
LM	Juvenile female	6

*Male that was peripheral to the group and attempting immigration.

M group had a relatively predictable daily range due to their use of only three sleeping sites, two of which were at the same end of their range. We arranged six feeding platforms (wooden tables, 0.75 m high, with a square flat top 0.75 x 0.75 m in size, Fig 1A and 1B) in a Z-shaped experimental array between M group’s sleep sites. The distances between the centers of the platforms were measured precisely and flagged stakes were placed in the ground beneath each platform to ensure that they were not moved between trials. With six sites to be visited in the array, there were 720 possible routes (6!). The group normally passed by the platforms twice per day and trials were carried out each day, whenever the monkeys ranged past the platforms (mean number of trials per day: 30.3; Range: 5–57). The arrangement of the platforms remained the same throughout the experiment so that the animals making routing decisions had prior knowledge of the layout and distance between sites. The Z-shaped array allowed differentiation of the shortest path and routes consistent with the nearest-neighbor rule (NNR) and the convex hull heuristic from all starting points but the central ones, where the shortest path and the convex hull were the same (platforms 3 and 4, Tables 2 and 3).

**Table 2:** Frequency and percentage of all paths taken during complete trials by single foraging vervet monkeys.

Number	Route	Heuristic*	Dist. (m)	Frequency	% Used
1	123456	SH	16.94	63	10.24
2	123465		17.49	16	2.6
3	132456		20.19	4	0.65
4	134256		24.67	1	0.16
5	134265		23.73	1	0.16
6	134562	NNR	21.49	73	11.91
7	134652		22.98	8	1.30
8	143256		23.75	1	0.16
9	143265		22.81	2	0.33
10	145632	CH	21.14	1	0.16
11	165432		21.88	1	0.16
12	213456	SH	17.49	27	4.39
13	213465		18.04	1	0.16
14	231456		19.82	2	0.33
15	234156		22.81	1	0.16
16	234561	NNR	21.88	100	16.26
17	234651		21.49	17	2.76
18	236541	CH	21.14	1	0.16
19	256431		22.98	1	0.16
20	321456	SH/CH	19.92	2	0.33
21	324561		25.23	1	0.16
22	341265		23.46	1	0.16
23	342156		23.83	1	0.16
24	345612		22.53	1	0.16
25	345621	NNR	21.59	12	1.95
26	346512		22.14	1	0.16
27	346521		23.08	2	0.33
28	431256		23.08	1	0.16
29	431265		22.14	1	0.16
30	432156	NNR	21.59	21	3.41
31	432165		22.53	6	0.98
32	432561		27.47	2	0.33
33	432651		25.59	2	0.33
34	435612		24.77	2	0.33
35	435621		23.83	2	0.33
36	453216		25.23	1	0.16
37	456231		22.6	1	0.16
38	456321	SH/CH	19.92	5	0.81
39	465123		23.25	1	0.16
40	513426		27.73	1	0.16
41	541326		23.82	1	0.16
42	543126		21.49	13	2.11
43	543216	NNR	21.88	111	18.05
44	543261		25.88	1	0.16
45	543612		23.75	1	0.16
46	543621		22.81	2	0.33
47	546312		20.37	1	0.16
48	563421		21.05	1	0.16
49	564312		18.04	1	0.16
50	564321	SH	17.49	36	5.85
51	634512		22.81	1	0.16
52	643125		22.98	3	0.49
53	643215	NNR	21.49	21	3.41
54	654312		17.49	7	1.14
55	654321	SH	16.94	27	4.39
Total				615	100

*SH = shortest path, NNR = nearest neighbor rule, CH = convex hull.

**Table 3:** Use of shortest route versus those paths that corresponded to a heuristic in examined trials (*n* = 500) and route distance.

Shortest Path (No Heuristic)			Convex Hull Heuristic			Nearest-Neighbor Rule		
Route	Dist. (m)	% Used	Route	Dist. (m)	% Used	Route	Dist. (m)	% Used
123456	16.94	12.6	123654	19.92	0	134562	21.49	14.6
			145632	21.14	0.2			
213456	17.49	5.4	236541	21.14	0.2	234561†	21.88	20
			214563	21.79	0			
321456*	19.92	0.4	321456*	19.92	0.4	345621	21.59	2.4
			365412	21.79	0			
456321*	19.92	1	456321*	19.92	1	432156	21.59	4.2
			412365	21.79	0			
564321	17.49	7.2	541236	21.14	0	543216†	21.88	22.2
			563214	21.79	0			
654321	16.94	5.4	654123	19.92	0	643215	21.49	4.2
			632145	21.14	0			
Total		32%			1.8%			67.6%

*Route consistent with the shortest path and the convex hull

†Route consistent with the ISS

M group had been the subject of several foraging experiments in the same location with the same platforms [11,32,33], so they were quickly habituated to again receive food rewards at the site. Once the platforms were set up in the array, we baited them with a single slice of peeled banana in the middle of each platform, so that individuals could grab the reward and eat it quickly before moving to the next target. We began recording data on the first day that the platforms were set up (Aug. 10, 15), as soon as the first monkeys arrived, because we were interested in their initial path choices and how later path choices may vary with experience. Platforms were re-baited to start another trial when all monkeys were ≥20 m away and the entire sequence could be re-baited before any individual could return. During each trial, a single observer (EAS) recorded the identity of vervets that approached the platforms and the sequence of events for each trial, including the order that sites were visited and which individual received the rewards. When multiple individuals arrived at the experimental array, food competition occurred. Here, we only examine trials where a single, identified individual went through the entire array and no food competition occurred (Table 2). These were trials with individuals that had run ahead of (or lagged behind) the rest of the group or those that could exclude other foragers from the platforms due to high dominance rank (Table 1). Experimental trials were conducted on public land and all experimental methods were carried out in accordance with relevant guidelines and regulations and were approved the McGill University Animal Care Committee (Protocol # 5061), the Uganda Wildlife Authority (Permit # UWA/TDO/33/02), and the Uganda National Council for Science and Technology (Permit # NS537).

### Data analyses

In total, 848 trials were run for this experiment. On 197 of these trials, two or more vervets acquired food from the platforms, so these competitive trials are not analyzed here. A single, identified individual visited all the platforms on 631 trials (hereafter “all solitary trials”) and revisits to empty platforms occurred during 16 trials (2.5%). We analyzed route optimization and heuristic use on the remaining 615 trials where a single, identified individual visited each platform once (hereafter “complete trials”). We tallied the number of routes used by the vervets in complete trials (Table 2) and used a chi-square test of homogeneity to determine whether each route was used with equal frequency. Wilcoxon signed-rank tests were used to examine whether the NNR was used significantly more often than the shortest path by the 17 individuals in complete trials (Table 1) and to determine whether individuals used paths consistent with the ISS more than other paths.

To examine the effect of experience on path choice we ran three analyses. First, a linear mixed-effects model was used to determine whether there was an influence of experience on the distance traveled through the route. The response variable was distance and fixed effects were trial number for each individual and subject ID. The starting platform and age-sex class were included as random factors in the model. For this analysis, all solitary trials including trials with revisits were used. Significance was determined with a likelihood ratio test that compared the model to a null model that included ID and the random effects. Second, we used a binary logistic regression to examine whether the use of paths consistent with the most frequently used heuristic (i.e., the NNR) changed with experience for individuals over the first 30 trials. Finally, to determine whether individuals had a tendency to repeat visitation sequences from the same starting platform as they gained experience in the route we used a similarity index on all solitary trials. Since vervets did not always start and end at the same point, we modified the similarity index (SI) described in Saleh and Chittka [34] for bumblebees. For each individual in each solitary trial after their first, the SI was calculated as the proportion of platforms that were visited in the same order as the trial before. As in Saleh and Chittka [34], an SI value of 0 means that sequences were completely different and a value of 1 means that they were the same; thus, higher values indicate greater congruence with the trial before. For each individual and each starting point that had a large enough sample size, we examined whether individuals had a significant positive correlation between SI and trial number (i.e., experience) using Spearman correlations. We also used Spearman correlations on the distance traveled from each starting point for two particular individuals to examine further how they may have optimized their routes with experience.

To examine the economics of path use relative to costs and rewards for vervets, first a Spearman correlation was used to determine whether there was a correlation between the ratio of the use of the NNR to the shortest path for each starting point in the array and the difference in distance between these two paths. In addition, a logistic regression was run for each starting point in the route (six tests) to determine if path use was predicted by the reward ratio obtained (# banana slices/distance traveled in m). All statistics were carried out in R version 3.3.2 [35] and the LMMs were carried out using the lme4 package (version 1.1–12) [36]. Tests were two-tailed with an alpha level of 0.05 set for significance.

## Results

### Use of the shortest path versus paths consistent with a heuristic

In complete trials with a single forager (*n* = 615), vervets used 55 different routes out of a possible 720 (Table 2). These 55 routes were not used with equal frequency (Chi-square, *χ*^*2*^ = 2638.05, *df* = 54, *P* <0.00001). Vervets took the overall shortest path 26% of the time (*n* = 160/615) and longer paths 74% of the time (455/615) (Table 3). The paths they took were consistent with an examined heuristic (the convex hull or NNR) in 56.4% of trials (*n* = 347/615). The convex hull heuristic was slightly more efficient than the NNR, leading to paths on average 0.67 m shorter, but vervet paths were rarely consistent with this heuristic (1.5% of complete trials, 1.8% of the 500 trials that were shortest or consistent with a heuristic, Table 3, hereafter “examined trials”). Paths were most often consistent with the slightly less efficient NNR (54.9% of complete trials, 67.6% of examined trials, Table 3).

**Fig 2:**
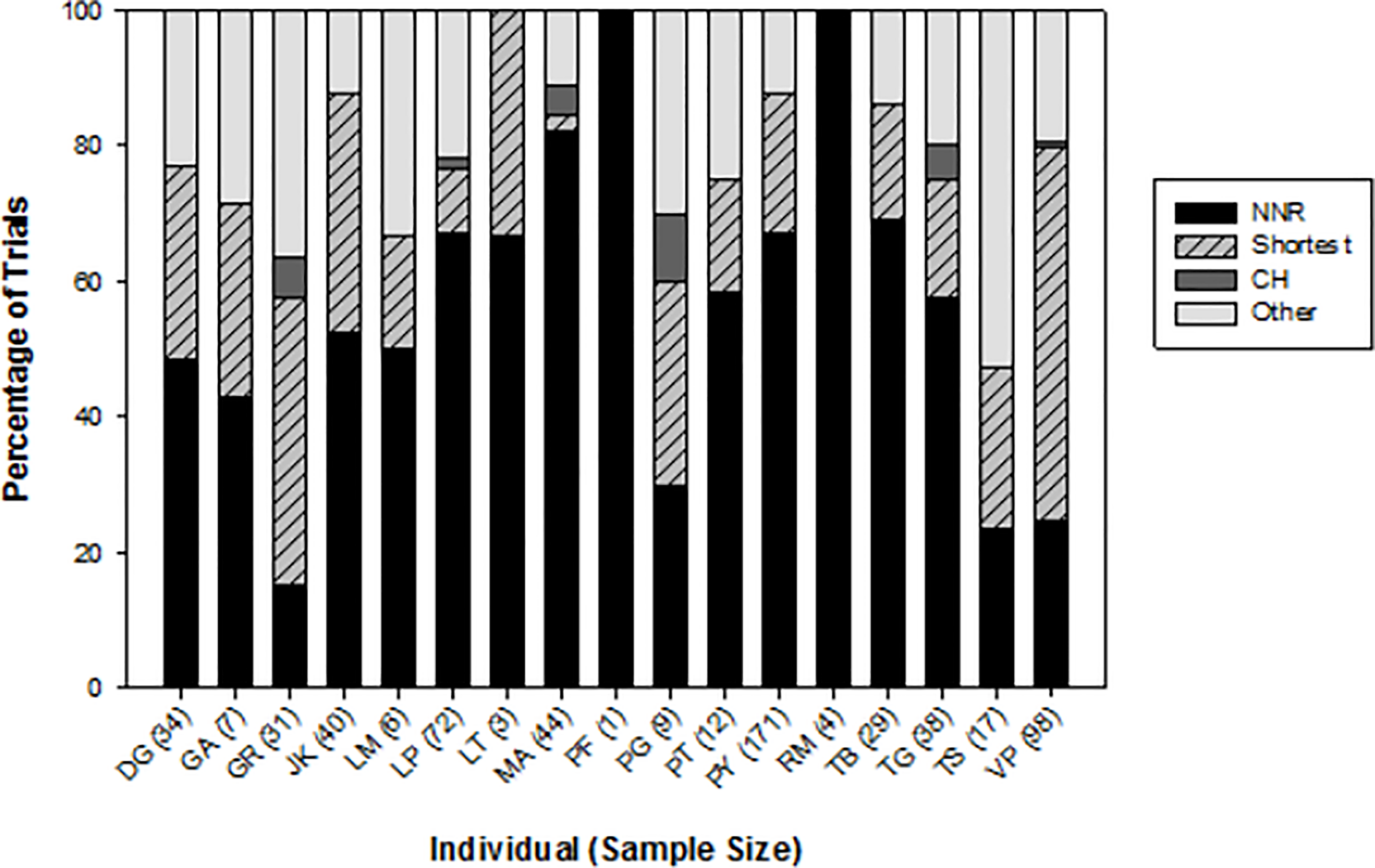
Percentage of paths consistent with each strategy for individual vervets with their sample size of trials in parentheses. The NNR was most common for 14 of 17 vervets.

**Fig 3:**
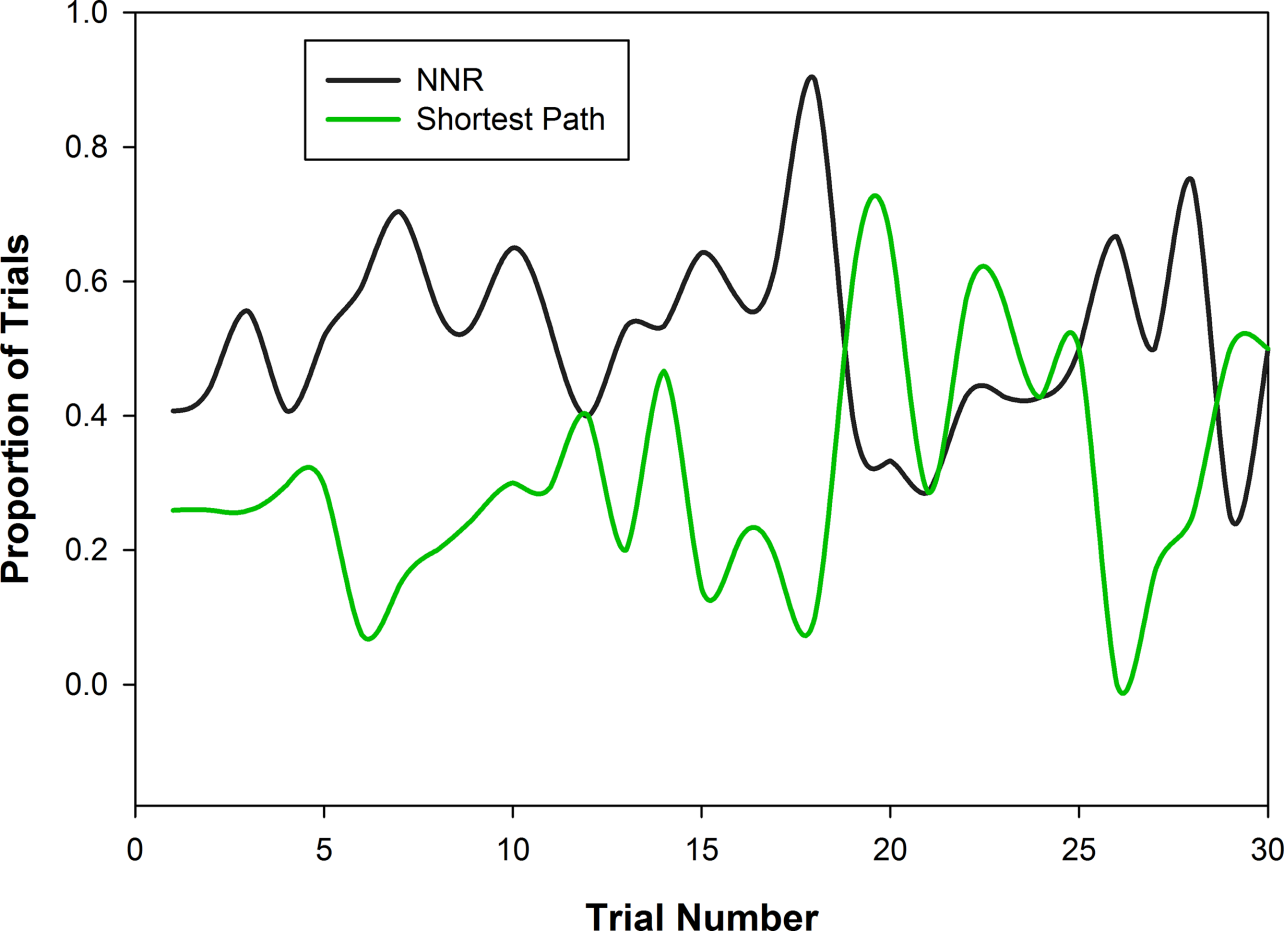
The proportion of paths consistent with the NNR and the shortest path as individuals gained more experience with the array.

During complete trials, a significant proportion of individual vervets (14/17) used paths consistent with the NNR more often than the shortest route (Wilcoxon signed-rank test, *n* = 17, *Z* = 2.37, *P* = 0.018, Fig 2). Two individuals took the shortest path most often (VP—55.1% (54/98), GR– 42.4% (14/33)) and one very old female (TS) used paths that were not consistent with any examined route or heuristic most often (52.9% of trials, 9/17) (Fig 2). There was no effect of experience for individuals on distance traveled through the route in all solitary trials (Linear mixed-effects model, Estimate = 0.004, *SE* = 0.002, likelihood ratio test, *χ*^*2*^(1) = 2.5*P* = 0.114). The first run through the array for each individual showed a mix of strategies, with paths consistent with the NNR most frequent and those consistent with the convex hull not used at all (*n* = 17, NNR 52.9%, Shortest path 23.5%, Other 23.5%). Use of the NNR did not vary with increasing experience over the first 30 trials for individuals (Binary logistic regression, Estimate = 0.002, *SE* = 0.013, *P* = 0.882, Fig 3). Individual analyses of the tendency to repeat visitation sequences showed mixed results. For the most part, individuals did not repeat visitation sequences more often as they gained experience with the route, with the exception of three (of 10) monkeys starting at platform 5 (Table 4). For these individuals, starting at this platform at the corner of the Z lead to more fidelity to a certain route. For two of these three individuals (LP and PY) this was the sub-optimal route straight up the line of platforms (discussed below for the ISS) and for the other individual (VP) this was the optimal route that he discovered with experience and stuck to from trial 18–23 (see discussion of this individual below).

**Table 4:** Correlations between similarity indices (SI) of successive trials and experience from each starting point for individuals.

Individual	Starting Platform	Trial Number	*r*_*s*_	*P*
DG	1	6	0.135	0.796
	2	8	-0.166	0.693
	5	18	-0.299	0.399
GR	1	16	-0.014	0.961
	5	6	-0.093	0.859
JK	2	9	0.52	0.146
	5	19	-0.035	0.883
LP	1	26	0289	0.152
	2	19	-0.387	0.102
	5	9	0.718	0.026*
	6	8	0.153	0.716
MA	1	18	-0.239	0.342
	2	10	0	N/A
	4	7	0.567	0.174
PY	1	37	-0.006	0.968
	2	26	-0.169	0.409
	4	15	0.4	0.138
	5	72	0.243	0.04*
	6	15	0.449	0.093
TB	2	15	-0.037	0.899
TG	1	13	-0.093	0.762
	6	8	0.126	0.765
TS	5	8	0.303	0.462
VP	1	30	0.19	0.316
	2	35	-0.02	0.905
	5	23	0.449	0.032*
	6	9	-0.183	0.636

*Significant result

### Importance of the first movement decision

**Fig 4:**
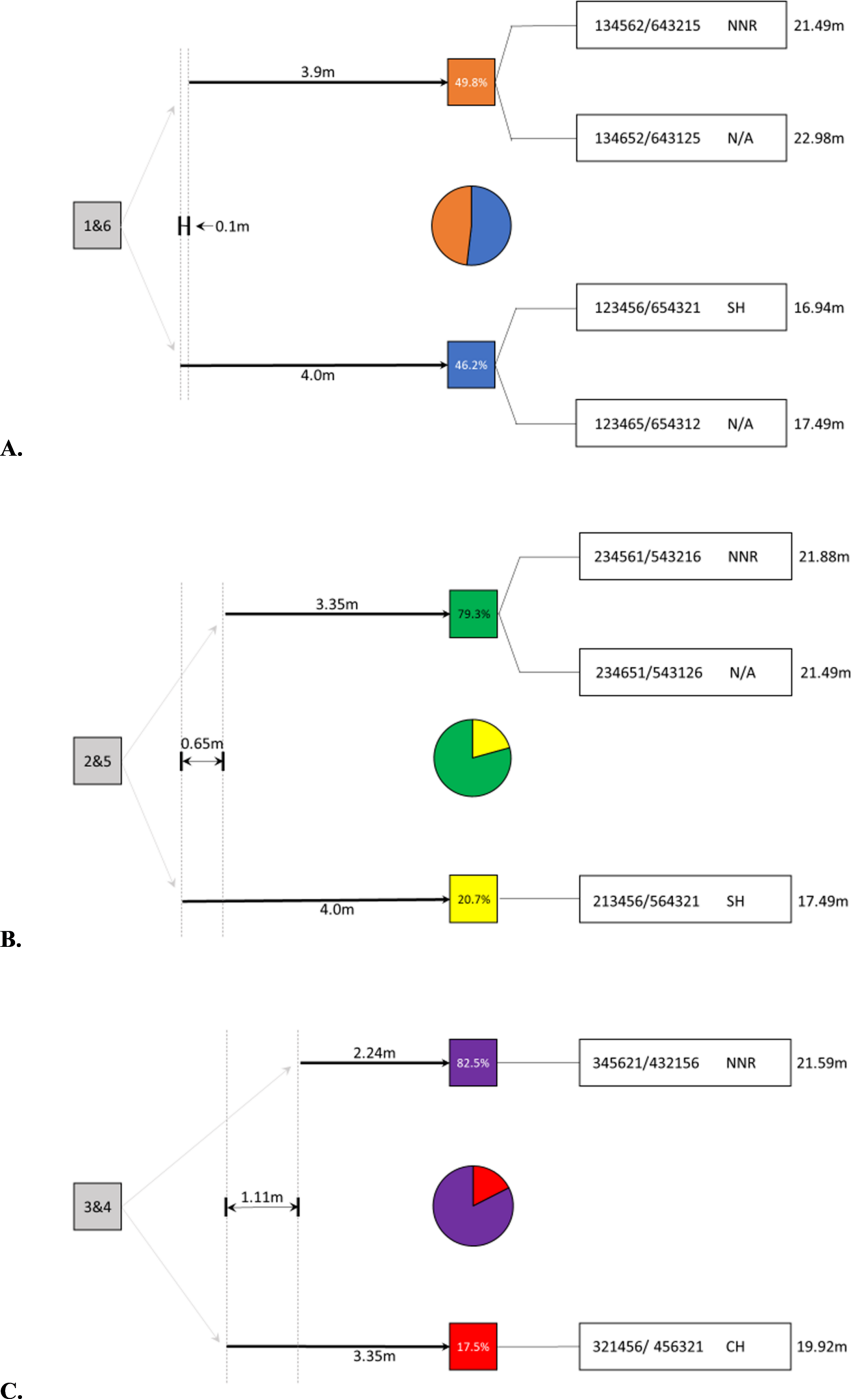
Similarities in vervet behavior from starting points on geometrically identical sides of the Z-array—showing the importance of the first decision in overall path efficiency. **(A)** From platforms 1 and 6, the next two closest platforms were almost equal in distance (0.1 m difference) and the vervets chose each in similar proportion. Grey pie portions show decisions other than the ones depicted. Choice of the slightly closer platform led to overall longer paths, while choice of the platform slightly further away led to more efficient paths. **(B)** From platforms 2 and 5, the next two closest platforms differed in distance by 0.65 m, and the closest was chosen 75.5% of the time, leading to inefficient overall paths. **(C)** From platforms 3 and 4, the next two closest platforms differed in distance by 1.11 m, and the closest was chosen 60.6% of the time, leading to inefficient overall paths. The ten most commonly used routes are displayed.

The Z-array was identical from either direction of approach, so decisions on one end were congruent with those on the other end. Thus, to examine how the first decision impacted the overall route taken by the vervets, we lumped data from starting points on either end, which led to the same geometric layout of platforms (1 lumped with 6, 2 with 5, 3 with 4) to choose from. This analysis showed that when the distances to the second choice of platforms were obviously different, the animals chose the shortest distance more often (Fig 4). However, from the tails of the Z (platforms 1 and 6) the distances to the next closest platforms were very similar (1–3, 6–4 = 3.9 m, 1–2, 6–5 = 4 m) and the animals chose their first movement from platforms 1 and 6 relatively equally between these options (Fig 4A), showing that they likely could not differentiate between these distances. This first decision in the array was critical as it determined whether the individual ended up taking an efficient or an inefficient route overall. If the individual chose the path with the shorter distance to the next platform first (moving from 1 to 3 or 6 to 4), they ended up taking a longer path through the array. However, if they made the decision to move the longer distance first (moving from 1 to 2 or from 6 to 5), they could take an optimal path through the array.

### Economics of vervets path choice

The ratio of complete paths consistent with the NNR to those that were shortest from each starting point in the Z-array was negatively correlated to the difference in distance between the two paths (Spearman, *n* = 6, *r*_*s*_ = -0.956, *P* = 0.0028). In other words, when the NNR was relatively efficient, paths were often consistent with it but when it was costlier in terms of distance, the use of the shortest path increased precipitously. To further examine the economics of vervet path choice from their point of view, we ran logistic regressions for each starting point in the route to determine if path use was predicted by the reward obtained over the distance traveled (“reward ratio” = 6 banana slices/distance traveled in m). This reward ratio varied depending on the route taken and was found to be a significant predictor of path use from every starting point in the array except platform 2 (Table 5), where 100 of 151 trials conformed to the less rewarding NNR because the monkeys had a strong tendency to move from the corner of the Z straight up the line to the opposite corner (see below, Fig 1A). Thus, even though the shortest path was not taken most frequently from any starting point except platform 6, the vervets used relatively shorter paths more often than longer routes.

**Table 5:** Effect of reward ratio as a predictor of path use from each starting point.

Starting Platform	*n* Trials	Chi-Square	Co-efficient (Std. Error)	*P*
1	172	129.19	23.68 (2.08)	<0.00001
2	151	3.71	-6.32 (3.53)	0.054
3	21	4.74	27.43 (12.56)	0.03
4	45	16.76	30.21 (7.51)	<0.00001
5	169	25.57	10.83 (2.14)	<0.00001
6	59	17.61	15.25 (3.73)	<0.00001

### Are vervets using an initial segment strategy (ISS)?

This experiment provided the vervets with a straight line of four reward platforms from one end of the Z-array to the other. Thus, if the monkeys used an ISS, it could be expected that, when they began the route at the end of this line, they should have moved straight through the line before going to collect the rewards on other platforms. Taking this type of route meant that the animal left behind one reward platform, and had to travel all the way back, crossing its original path, to go get it; making these paths quite inefficient and leaving behind a reward that could potentially be taken by a competitor. Nonetheless, in the 319 complete trials where monkeys began the route at the corner of Z-array at the end of this line (platforms 2 or 5), they traveled straight up the line of four platforms 212 times (66.5% of trials). Twelve individuals had a sample size of at least five complete trials starting at platforms 2 and 5, and of these, nine monkeys took the initial straight line more often than other paths, however this was not significant (Wilcoxon signed-rank test, *n* = 12, *W* = 41, *P* = 0.112). Notably, two of the three monkeys that did not take the straight path immediately most often were also the two that found the shortest path consistently (VP and GR). When these animals are removed from the sample, most of the remaining monkeys took the initial straight segment more often than any other route from these starting points (*n* = 10, *W* = 51, *P* = 0.01) and the percentage use of paths consistent with an ISS when starting at platforms 2 or 5 jumped to 74.3% (188/253). The paths that led straight up the line of the Z from platforms 2 and 5 were also consistent with the NNR, so use of an ISS could help explain why the NNR was so prevalent in this array.

### Evidence that some individuals considered the whole array

**Fig 5:**
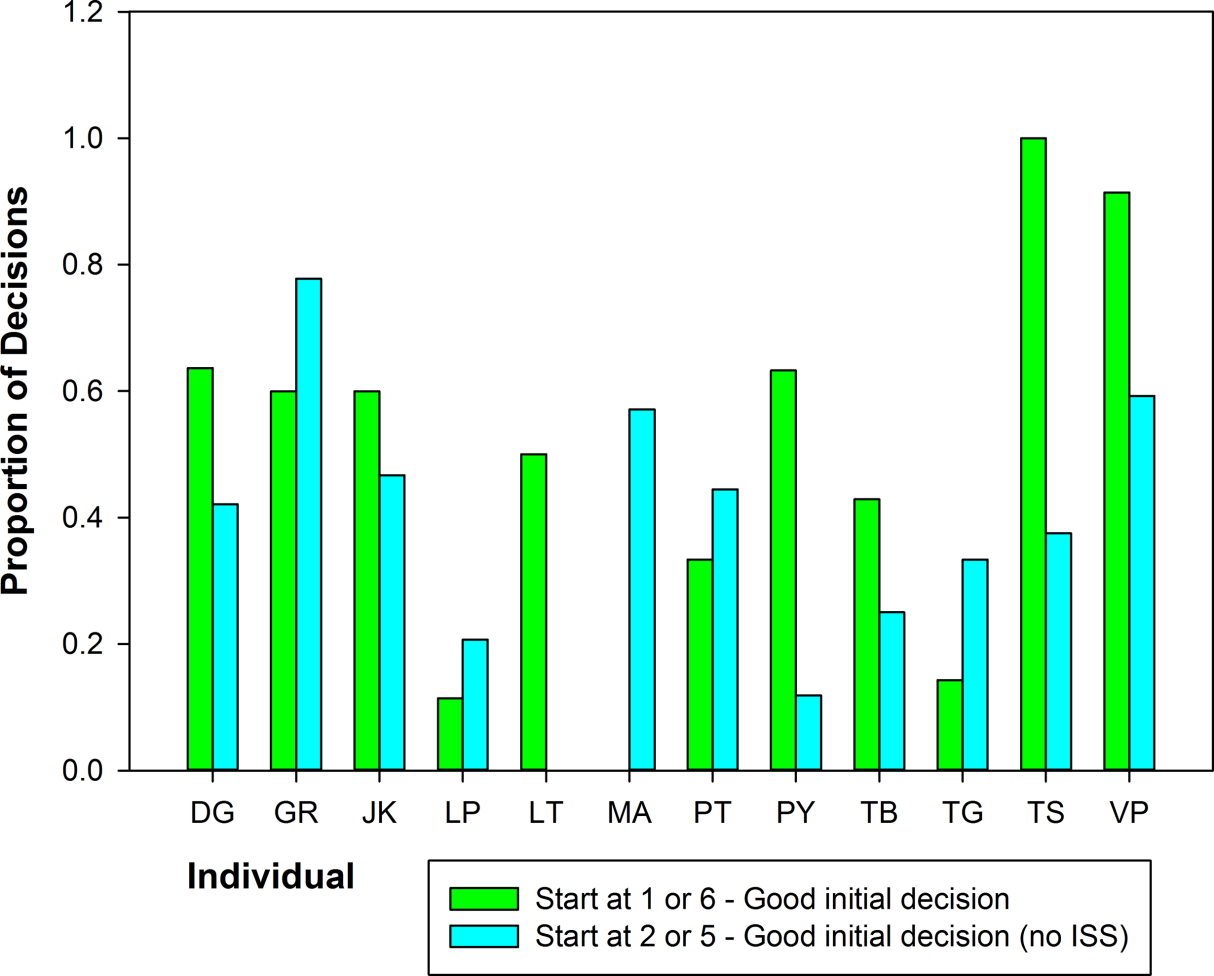
Individual differences in good decisions for optimally completing the rest of route. Adult female GR and adult male VP were more successful than other individuals in solving the route and they show more initial good decisions when starting the route at various platforms than other monkeys.

We investigated the success of adult female GR and adult male VP relative to other monkeys further, by looking at their decisions at different points in the array. VP (*n* = 98 complete trials) in particular was good at choosing the longer distance initially (4 m versus 3.9 m) from platforms at the end of the Z (1 and 6), which set him up to take the shortest path over the whole route (Fig 5). GR (*n* = 31 complete trials) was less consistent in making this good initial decision from 1 and 6 than VP, but she was still better than most other monkeys. GR showed the best performance of all individuals in resisting moving up the straight line of platforms when starting at platforms 2 and 5 (i.e., the ISS) and therefore she more often took the shortest path when starting at these platforms (Fig 5). VP used paths consistent with an ISS more than GR when starting at the end of the straight line (platforms 2 and 5) but still far less than other monkeys. Taken together, these data suggest that GR and VP both often took into consideration all of the platforms in the array when making their movement decisions.

Though the earlier LMM showed that, generally, the monkeys did not use shorter routes as they gained experience, we also extracted GR and VP’s data to look at the effect of experience on their distance traveled in the array. The sample size was large enough for GR when she began at platform 1 and at platform 5 and showed that she did not become more efficient with experience (Platform 1: *n* = 17, *r*_*s*_ = 0.004, *P* = 0.984; Platform 5: *n* = 7, *r*_*s*_ = 0.139, *P* = 0.764), since she often used the shortest path, even early on. VP, however, did show evidence of optimizing over time when starting on two of four platforms with enough trials (Platform 1: *n* = 31, *r*_*s*_ = -0.439, *P* = 0.014; Platform 2: *n* = 36, *r*_*s*_ = -0.171, *P* = 0.319; Platform 5: *n* = 23, *r*_*s*_ = -0.272, *P* = 0.197; Platform 6: *n* = 10, *r*_*s*_ = -0.731, *P* = 0.016). From Platform 1 and 6, the shortest path was used more often as he gained experience, which suggests that he was learning over time.

Overall, if data from GR and VP are removed from complete trials, the use of paths consistent with heuristic strategies (NNR or convex hull) goes up from 56.4% to 64.9% (315/485). This is due to an increase in the use of paths consistent with NNR (up from 54.9% to 63.7%). Paths consistent with the convex hull drop slightly (from 1.46% to 1.24%) with the removal of GR and VP’s data, because there are instances from the central platforms (3 and 4) where the convex hull is consistent with the shortest path.

## Discussion

This study allowed us to answer several lingering questions about how vervets are solving multi-destination routes. In the Z-array we used, the shortest path always differed from paths consistent with the NNR and was also usually different from those consistent with the convex hull. Thus, we were able to show that most vervet monkeys did not consistently find the shortest path through the array. Though early work by Cramer and Gallistel suggested that vervets might be particularly good at planning their paths through a multi-destination route [9], this study shows that the individuals in our sample typically took longer paths. The vervets in this study only took the optimal path 26% of the time and the effect of spatial scale must be considered. Previous research on bumblebees found that the longer the distances that bees had to fly in an array, the faster they were at optimizing their routes [17], which suggests that the low rate of optimization for vervets could be due to the small spatial scale of this experiment. However, our previous research argues against this interpretation. We have found that even though the vervets will run hundreds of meters to participate in our experiments, they are remarkably efficient moving through the array once they arrive. For instance, on an easy-to-solve pentagon array with sites five meters apart, the monkeys took the shortest path 91% of the time [33]. Despite only taking the most efficient route a quarter of the time in the Z-array, the vervets behavior was usually determined by the rewards obtained per meter traveled, which shows that they did behave economically overall. Similarly, in a foraging experiment with a group of wild capuchin monkeys (*Cebus apella nigritus*), Janson [1] also found that the monkeys use of a detour within a multi-destination route could be best predicted from the rewards obtained at this site relative to the costs of travel.

Our data did show important individual variation in success at solving the SPP and suggest that at least some individuals plan their routes. Two monkeys, adult female GR and adult male VP, had much greater success finding the shortest path relative to the other individuals in the sample. For VP, this stemmed mostly from making a good initial decision to choose the second closest platform, rather than the closest one when starting the route at the ends of the Z and apparent learning with experience which routes were shorter. For GR, success was mostly due to avoiding moving straight up the line of platforms available from the corners of the Z and instead turning and getting the resource that would be left behind at the tail of the Z before moving on. These behaviors suggest that these two individuals considered the layout of the whole array before making their initial decisions and planned their routes. Though not without controversy [37,38], strong evidence for planning ahead has been found for several animal species [39–43]. Particularly relevant to this study is the finding that, when given spatial information about the location of nectar, and thus the potential to plan, noisy miner birds (*Manorina melanocephala*) make far fewer search errors than when not given spatial information [43]. This study on miner birds was the first to find planning ahead in an animal that did not also include a disassociation from the current motivational state, leading the authors to suggest that planning of foraging routes may be the most cognitively rudimentary type of planning [43]. Here, we provide an additional example of this type of planning.

The consistent location of feeding platforms in our Z-array certainly gave the vervets plenty of time to plan their routes, so why did only two individuals show strong evidence of planning? We argue that our data actually do suggest that other monkeys are capable of planning their routes. Our finding that the use of paths consistent with the NNR dropped off precipitously, and use of the shortest path increased sharply, when the NNR was less efficient shows that it may be the cognitive cost of this planning which keeps heuristic use so high. Vervets, other than GR and VP, may have been able to find optimal solutions to the SPP but only did so when easy-to-use heuristics no longer led to relatively efficient routes. GR and VP may have either perceived a higher efficiency cost to heuristic use than the other monkeys or they had to bear a lower cognitive cost to find the shortest path. For most monkeys in this study, repeated use of the NNR was accurate enough in most cases and not too costly, a case of satisficing over maximizing [44,45].

The frequent use of paths consistent with the NNR by most monkeys in this study suggests that vervets, just like humans, come to relatively efficient paths in multi-destination routes by applying simple rules-of-thumb [5,20]. If humans and vervets both use heuristics to solve multi-destination routes, it is an interesting question as to whether these species converged separately on the use of certain rules-of-thumbs (i.e., the NNR and the convex hull [11]) or whether these could be part of a mental toolkit (the “adaptive toolkit” [46]) shared by a common ancestor or perhaps shared by all animals [47].

By providing a less circular route geometry to the vervets compared to a previous study [11], we showed a much lower frequency of paths consistent with the convex hull heuristic. Even though use of the convex hull heuristic in the Z-array would have led to shorter and more efficient paths than those provided by the NNR (on average 0.67 m shorter), monkeys very rarely used these paths (1.8% of examined trials). This makes sense if animals are selected to solve certain path problems based on their foraging strategy. As multiple central place foragers [8] with several different sleeping sites, vervets should be selected to solve SPPs, rather than TSPs, for which the convex hull is often ideal [5]. These data suggest that the high frequency of paths consistent with the convex hull in Teichroeb [11], could potentially be explained by a different mechanism or a heuristic not investigated. Here, we found evidence that, like humans solving SPPs where they have to move to a distant point, vervets may also minimize cognitive load by avoiding turning until it becomes necessary, thus taking an ISS [30]. Given this support for the ISS as well as other evidence of use of the NNR in this array, we propose that the vervets may be using a heuristic rule to solve SPPs that is a combination of these mechanisms, conceptualized as “*If there is a straight route*, *take it*. *If not*, *go to the next nearest resource*”. We term this the “region heuristic” because it is consistent with what humans do when moving between distant points or traveling through complex paths, which is plan their routes on a region-by-region basis [48–50]. People often take different paths from A to B, compared to from B to A, because they appear to break down the route into segments, following straight lines until they are forced to turn at a particular juncture [27,30]. We could not test this new “region heuristic” with the current Z-array because routes consistent with ISS and the NNR were usually identical. However, in the array used in Teichroeb [11], a rule like this may have explained the results and the high frequency of use of routes consistent with the convex hull, though it depends on what the vervets perceive as a straight line. The platforms along the edges of that array were slightly offset and the geometry of objects is known to impact animal navigation, influencing axes of orientation and determining the direction of travel in some cases [51,52]. Thus, moving on from this research, our future work will test the “region heuristic” with an experiment specifically designed to provide clear answers. In addition, we will manipulate the cost of using this heuristic by changing the distance between platforms. This approach will help confirm our supposition here that most vervets can plan their routes when cognitive minimizing strategies are no longer beneficial.

## Supporting information

S1 Table Data for all trials with no competition. (XLSX)

## References

[1] Janson CH. Experimental evidence for route integration and strategic planning in wild capuchin monkeys. Anim Cogn. 2007; 10: 341–356. doi: 10.1007/s10071-007-0079-2 17464518 10.1007/s10071-007-0079-2

[2] Lihoreau M, Chittka L, Raine NE. Trade-off between travel distance and prioritization of high-reward sites in traplining bumblebees. Func Ecol. 2011; 25: 1284–1292. 10.1111/j.1365-2435.2011.01881.x PMC3260656 22267886

[3] Janson CH. Death of the (traveling) salesman: primates do not show clear evidence of multi-step route planning. Am J Primatol. 2013; 76: 410–420. doi: 10.1002/ajp.22186 23934927 10.1002/ajp.22186

[4] Lawler EL, Lenstra JK, Rinooy Kan AHG, Schmoys DB. The Traveling Salesman Problem: A Guided Tour of Combinatorial Optimization. New Jersey: Wiley; 1985.

[5] MacGregor JN, Chu Y. Human performance on the traveling salesman and related problems: a review. J Prob Solv. 2011; 3: 1–29.

[6] Orians GH, Pearson NE. On the theory of central place foraging In: Horn DJ, Mitchell RK, Stairs GR, editors. Analysis of Ecological Systems. Ohio: Ohio University Press; 1979 pp 155–177.

[7] Schoener TW. Generality of the size-distance relation in models of optimal feeding. Am Nat. 1979; 114: 902–914.

[8] Chapman CA, Chapman LJ, McLaughlin RL. (1989) Multiple central place foraging by spider monkeys: travel consequences of using many sleeping sites. Oecologia 1989; 79: 506–511. doi: 10.1007/BF00378668 28313485 10.1007/BF00378668

[9] Cramer AE, Gallistel CR. Vervets as travelling salesmen. Nature 1997; 387: 464–464. doi: 10.1038/387464a0 9168107 10.1038/387464a0

[10] Lihoreau M. Chittka L, Le Comber SC, Raine NE. Bees do not use nearest-neighbour rules for optimization of multi-location routes. Biol Lett. 2012; 8: 13–16. doi: 10.1098/rsbl.2011.0661 21849311 10.1098/rsbl.2011.0661 PMC3259973

[11] Teichroeb JA. Vervet monkeys use paths consistent with context-specific spatial movement heuristics. Ecol Evol. 2015; 5: 4706–4716. doi: 10.1002/ece3.1755 26668734 10.1002/ece3.1755 PMC4670061

[12] Menzel EW. Chimpanzee spatial memory organization. Science 1973; 182: 943–945. doi: 10.1126/science.182.4115.943 17737534 10.1126/science.182.4115.943

[13] MacDonald SE, Wilkie D. Yellow-nosed monkeys’ (*Cercopithecus ascanius whitesidei*) spatial memory in a simulated foraging environment. J Comp Psychol. 1990; 104: 382–397.

[14] Bureš J, Burešováa O, Nerad L. Route selection by rats and humans in a navigational traveling salesman problem. Behav Brain Res. 2006; 52: 133–142. 10.1016/s0166-4328(05)80223-2 1294192

[15] Gibson BM, Wasserman EA, Kamil AC. Pigeons and people select efficient routes when solving a one-way “traveling salesperson” task. J Exp Psychol: Anim Behav Process. 2007; 33: 244–261. 17620024 10.1037/0097-7403.33.3.244

[16] Lihoreau M, Raine NE, Reynolds AM, Stelzer RJ, Lim KS, Smith AD, et al Radar tracking and motion-sensitive cameras on flowers reveal the development of pollinator multi-destination routes over large spatial scales. PLoS Biol. 2012; 10: e1001392 doi: 10.1371/journal.pbio.1001392 23049479 10.1371/journal.pbio.1001392 PMC3462218

[17] Reynolds AM, Lihoreau M, Chittka L. A simple iterative model accurately captures complex trapline formation by bumblebees across spatial scales and flower arrangements. PLoS Comput Biol. 2013; 9: e1002938 doi: 10.1371/journal.pcbi.1002938 23505353 10.1371/journal.pcbi.1002938 PMC3591286

[18] Gigerenzer G, Todd PM. Simple Heuristics that Make us Smart. UK: Oxford University Press; 1999.

[19] Golden BL, Stewart WR Jr. Empirical analysis of heuristics In: Lawler EL, Lenstra JK, Rinooy Kan AHG, Schmoys D. B., editors. The Traveling Salesman Problem: A Guided Tour of Combinatorial Optimization. New Jersey: Wiley; 1985 pp. 207–249.

[20] Weiner JM, Ehbauer NN, Mallot HA. Path planning and optimization in the traveling salesman problem: nearest neighbor vs. region-based strategies. Dagstuhl Sem Proceed. 2006; 05491: 1–21.

[21] Gross J, Zank C, Hobbs N, Spalinger DE. Movement rules for herbivores in spatially heterogeneous environments: responses to small scale pattern. Landsc Ecol. 1995; 10: 209–217.

[22] Pyke GH. Optimal foraging in hummingbirds: rules of movement between inflorescences. Anim Behav. 1981; 29: 889–896.

[23] Chronicle EP, MacGregor JN, Ormerod TC, Burr A. It looks easy! Heuristics for combinatorial optimization problems. Q J Exp Psychol. 2006; 59: 783–800. 10.1080/02724980543000033 16707362

[24] MacGregor JN, Chronicle EP, Ormerod TC. A comparison of heuristic and human performance on open versions of the Traveling Salesperson Problem. J Prob Solv. 2006; 1: 33–43.

[25] Layton OW, O’Connell T, Phillips F. The traveling salesman problem in the natural environment. J Vis. 2009; 9(1145): 10–1167.

[26] Ekstrom AD, Huffman DJ, Starrett M. Interacting networks of brain regions underlie human spatial navigation: a review and novel synthesis of the literature. J Neurophysiol. 2017; 11: 3328–2244. 10.1152/jn.00531.2017 PMC5814720 28931613

[27] Christenfeld N. Choices from identical options. Psychol Sci. 1995; 6: 50–55.

[28] Wiener JM, Mallot HA. Fine-to-course route planning and navigation in regionalized environments. Spatial Cogn Compu. 2003; 3: 331–358.

[29] Stern E, Leiser D. Levels of spatial knowledge and urban travel modeling. Geograph Anal. 1988; 20: 140–155.

[30] Bailenson JN, Shum MS, Uttal DH. The initial segment strategy: a heuristic for route selection. Mem Cogn. 2000; 28: 306–318. 10.3758/bf03213808 10790984

[31] Kingdon J, Gippoliti S, Butynski TM, De Jong Y. Chlorocebus pygerythrus. The IUCN Red List of Threatened Species 2008; e.T136271A4267738.

[32] Teichroeb JA, Chapman CA. Sensory information and associative cues used in food detection by wild vervet monkeys. Anim Cogn. 2014; 17: 517–528. doi: 10.1007/s10071-013-0683-2 24045849 10.1007/s10071-013-0683-2

[33] Teichroeb JA, Aguado WD. Foraging vervet monkeys optimize travel distance when alone but prioritize high-reward food sites when in competition. Anim Behav. 2016; 115: 1–10.

[34] Saleh N, Chittka L. Traplining in bumblebees (*Bombus impatiens*): a foraging strategy’s ontogeny and the importance of spatial reference memory in short-range foraging. Oecologica 2007; 151: 719–730. 10.1007/s00442-006-0607-9 17136553

[35] R Core Team. R Foundation for Statistical Computing, Vienna, Austria. URL http://www.R-project.org/; 2017.

[36] Bates D, Maechler M, Bolker B, Walker S. Fitting linear mixed-effects models using lme4. J Stat Softw. 2015; 67: 1–48.

[37] Suddendorf T, Corballis MC. Behavioural evidence for mental time travel in nonhuman animals. Behav Brain Res. 2010; 215: 292–298. doi: 10.1016/j.bbr.2009.11.044 19962409 10.1016/j.bbr.2009.11.044

[38] de Waal FBM, Ferrari PF. Towards a bottom-up perspective on animal and human cognition. Trends Cognitive Sci. 2010; 14: 201–207. 10.1016/j.tics.2010.03.003 20363178

[39] Naqshabandi M, Roberts WA. Anticipation of future events in squirrel monkeys (*Saimiri sciureus*) and rats (*Rattus norvegicus*): tests of the Bischof-Kohler hypothesis. J Comp Psychol. 2006; 120: 345–357. doi: 10.1037/0735-7036.120.4.34 17115855 10.1037/0735-7036.120.4.34

[40] Raby CR, Dickinson A, Clayton NS. Planning for the future by western scrub-jays. Nature 2007; 445: 919–921. doi: 10.1038/nature05575 17314979 10.1038/nature05575

[41] Osvath M, Osvath H. Chimpanzee (*Pan troglodytes*) and orangutan (*Pongo abelii*) forethought: self-control and pre-experience in the face of future tool use. Anim Cogn. 2008; 11: 661–674. doi: 10.1007/s10071-008-0157-0 18553113 10.1007/s10071-008-0157-0

[42] Visalberghi E, Spagnoletti N, da Silva EDR, Andrade FR, Ottoni E, Izar P. Distribution of suitable hammers and transport of hammer tools and nuts by wild capuchin monkeys. Primates 2009; 50: 95–104. doi: 10.1007/s10329-008-0127-9 19172379 10.1007/s10329-008-0127-9

[43] Sulikowski D, Burke D. Noisy miners plan ahead: cryptic signaling of reward location impairs search for nectar, but not for invertebrates. Anim Behav. 2015; 102: 149–155.

[44] Simon HA. A behavioral model of rational choice. Quant J Econ. 1955; 69: 174–183.

[45] Ward D. The role of satisficing in foraging theory. Oikos 1992; 63: 312–317.

[46] Gigerenzer G, Selton R. Bounded Rationality: The Adaptive Toolbox. Cambridge, MS: MIT Press; 2001.

[47] Hauser MD. Wild Minds. New York: Henry Holt and Co.; 2000.

[48] Bailenson JN, Shum MS, Uttal DH. Road climbing: principles governing asymmetric route choices on maps. J Environ Psychol. 1988; 18: 251–264.

[49] Downs RM, Liben LS, Daggs DG. On education and geographers: the role of cognitive development theory in geographic education. Ann Assoc Am Geograph. 1988; 78: 680–700.

[50] Sadalla EK, Burroughs WJ, Staplin LJ. Reference points in spatial cognition. J Exper Psychol. 1980; 6: 516–528. 7430967

[51] Cheng K. Reflections on geometry and navigation. Conn Sci. 2005; 17: 5–21.

[52] Benhamou S, Poucet B. Landmark use by navigating rats (*Rattus norvegicus*) contrasting geometric and featural information. J Comp Psychol. 1998; 112: 317–322.

